# IMPACT OF SOCIALIZATION IN LOW-INCOME URBAN ELDERLY COMMUNITIES

**DOI:** 10.1093/geroni/igz038.1944

**Published:** 2019-11-08

**Authors:** Taylor Wilkerson, Schanea Ward, Amy Popovich, Pamela Parsons, Faika A Zanjani

**Affiliations:** 1 Virginia Commonwealth, Richmond, Virginia, United States; 2 Richmond City Health District, Richmond, Virginia, United States

## Abstract

Older adults who experience social isolation have higher rates of mortality relative to their counterparts. Social interactions are an important way to combat this isolation. This research aims to better understand how social isolation in older adults living in low-income households in Richmond, Virginia (RVA) is related to their economic, physical, and psychological health status. As part of the VCU iCubed Health and Wellness Aging Core and in collaboration with the Richmond Memorial: East End Housing Coalition for Older Adults, older adults from a selected public housing unit (n=28) self-reported their financial status, experiences with physical and psycho-social health, and feelings of social isolation. Survey participants were 71.4% female, the mean age was 69.75 years, and 25% were high school graduates. Participants averaged 34 years living in the area and reported an average of $300 to spend on rent monthly. Overall, 55% (n=20) reported having two or more supports and 61% (n=22) reported hardly ever feeling isolated. However, a small subset of the sample reported having either no supports (5.6%, n=2) and 41.7% (n=15) lacked companionship some of the time or often. A one-way ANOVA was conducted and it was determined that participants who reported feeling left out more often were significantly more likely to report stress, anxiety, and depression (F[2, 25] = 6.998). Findings support the existence of supportive communities formed in low-income areas. Findings also indicate some older individuals residing in public housing in RVA experience social isolation, linking them to poorer psycho-social health.

